# Giant Intraparenchymal Splenic Cyst Causing Massive Splenomegaly: Case Report and Surgical Management

**DOI:** 10.7759/cureus.110502

**Published:** 2026-06-08

**Authors:** Christian Ballardo Medina, Jaime Matus Rojas, Rodolfo Lopez Hernandez, Mariana Burgueño ibarra, Jesus Antonio Velazquez Leon, Evelyn Gabriela Murillo Valdez

**Affiliations:** 1 General Surgery, Institute of Social Security and Services for State Workers (ISSSTE), Autonomous University of Sinaloa (UAS), Culiacán, MEX; 2 General Surgery, Institute of Social Security and Services for State Workers (ISSSTE), Autonomous University of Sinaloa (UAS), SINALOA, MEX; 3 General Surgery, Institute of Social Security and Services for State Workers (ISSSTE), Autonomous University of Sinaloa (UAS), Sinaloa, MEX

**Keywords:** intraparenchymal cyst, massive splenomegaly, non-parasitic splenic cyst, post-splenectomy vaccines, total splenectomy

## Abstract

Splenic cysts are rare entities, with a barely reported incidence of cases. Giant intraparenchymal cysts are exceptional and may present diagnostic and therapeutic challenges. We report the case of a 22-year-old male presenting with fever, weight loss, and abdominal symptoms. Imaging revealed massive splenomegaly secondary to a large intraparenchymal splenic cyst measuring approximately 13 cm. The patient underwent elective open splenectomy with an uneventful postoperative course. Histopathology confirmed an intraparenchymal splenic cyst.

## Introduction

Splenomegaly secondary to splenic cysts is a rare condition in which the spleen increases in size due to the presence of single or multiple cysts within the splenic parenchyma. These cysts may be congenital, post-traumatic, infectious, or parasitic in origin. Most cases are asymptomatic and are diagnosed incidentally; however, when they reach a significant size, they may produce symptoms related to compression of adjacent structures or displacement of the splenic parenchyma, such as left upper quadrant pain, early satiety, or a palpable mass.

Primary non-parasitic splenic cysts are a clinical rarity, representing approximately 30-40% of all splenic cysts, although together with post-traumatic cysts, they are among the most commonly reported cystic splenic lesions in the literature [1,2].

Currently, splenic cysts are considered a rare clinical condition with an incidence of approximately 0.07% in the general population. Primary cysts are subdivided into parasitic (60%) and non-parasitic cysts according to their etiology. Non-parasitic cysts are commonly congenital, usually presenting at a young age and typically located in the upper pole of the spleen [1]. Giant splenomegaly secondary to an intraparenchymal cyst is exceptionally rare, and its surgical management remains controversial.

## Case presentation

A 22-year-old male, with no relevant medical or surgical history, presented with intermittent fever up to 39.2 °C, predominantly nocturnal, associated with chills, asthenia, adynamia, myalgia, arthralgia, retro-orbital headache, nausea, and vomiting for 14 days before admission. Subsequently, the patient developed early satiety, diffuse abdominal pain, and an approximate weight loss of 10 kg in one month.

Laboratory studies revealed leukocytosis (>14.67 ×10³/mm³; reference range: 4.5-11.0) with neutrophilia (86.8%), as well as elevated liver enzymes, including aspartate aminotransferase (AST) of 45.5 U/L, alanine aminotransferase (ALT) of 70.6 U/L, and alkaline phosphatase (129 U/L) (Table 1).

**Table 1:** Laboratory results. AST: aspartate aminotransferase; SGOT: glutamic oxaloacetic transaminase; ALT: alanine aminotransferase; SGPT: glutamic pyruvic transaminase.

Test	Result	Reference values
Creatinine	0.59 mg/dL	0.6-1.3 mg/dL
Urea	25.2 mg/dL	10-50 mg/dL
Bun	11.78 mg/dL	7-18 mg/dL
AST (SGOT)	45.5 U/L	15-40 U/L
ALT (SGPT)	70.6 U/L	17-40 U/L
Alkaline phosphatase	129 U/L	40-130 U/L
Albumin	2.62 g/dL	3.5-5 g/dL
White blood cells	13.23 x 10^3^/mm^3^	4.5-11 x 10^3^/mm^3^
Platelets	229 x 10^3^/U^3^	150-400 x 10^3^/U^3^

**Figure 1:**
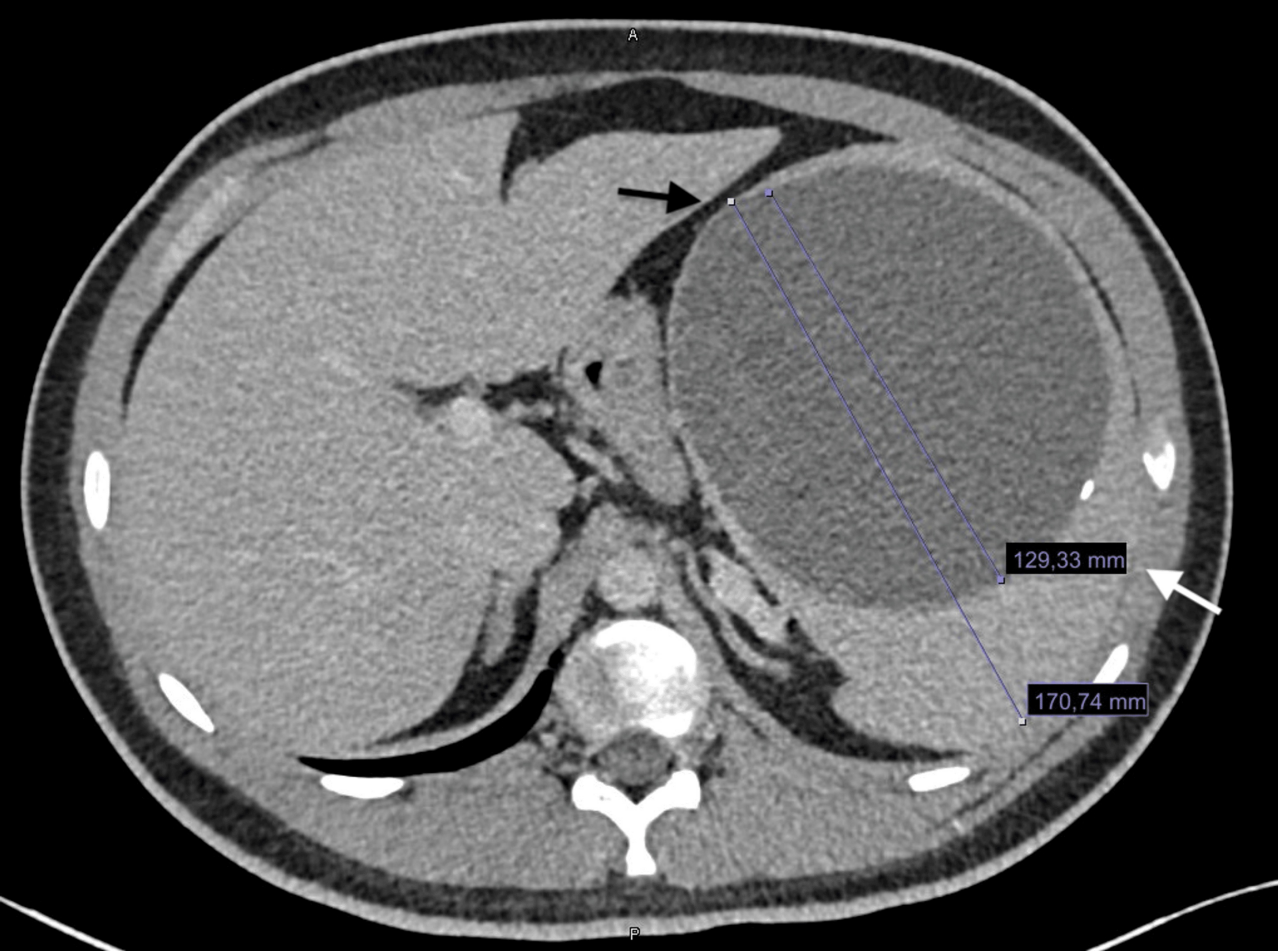
Contrast-enhanced computed tomography (axial view). Splenic measurement of 170.74 mm (black arrow). Splenic cyst measurement of 129.33 mm (white arrow).

**Figure 2:**
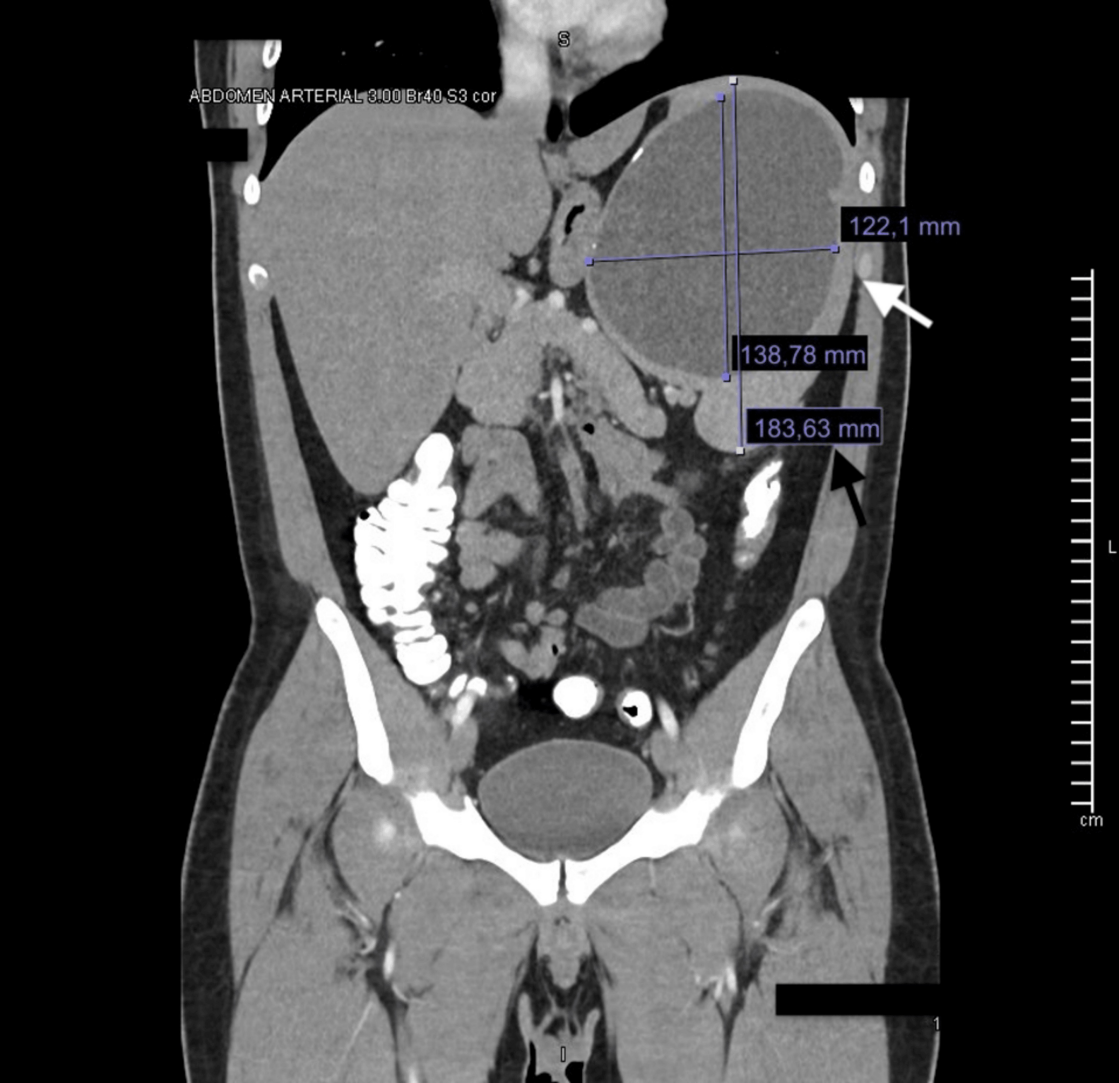
Contrast-enhanced computed tomography (coronal view). Splenic measurement of 183.63 mm (black arrow). Splenic cyst measurement of 122.1 × 138.78 mm (white arrow).

**Figure 3:**
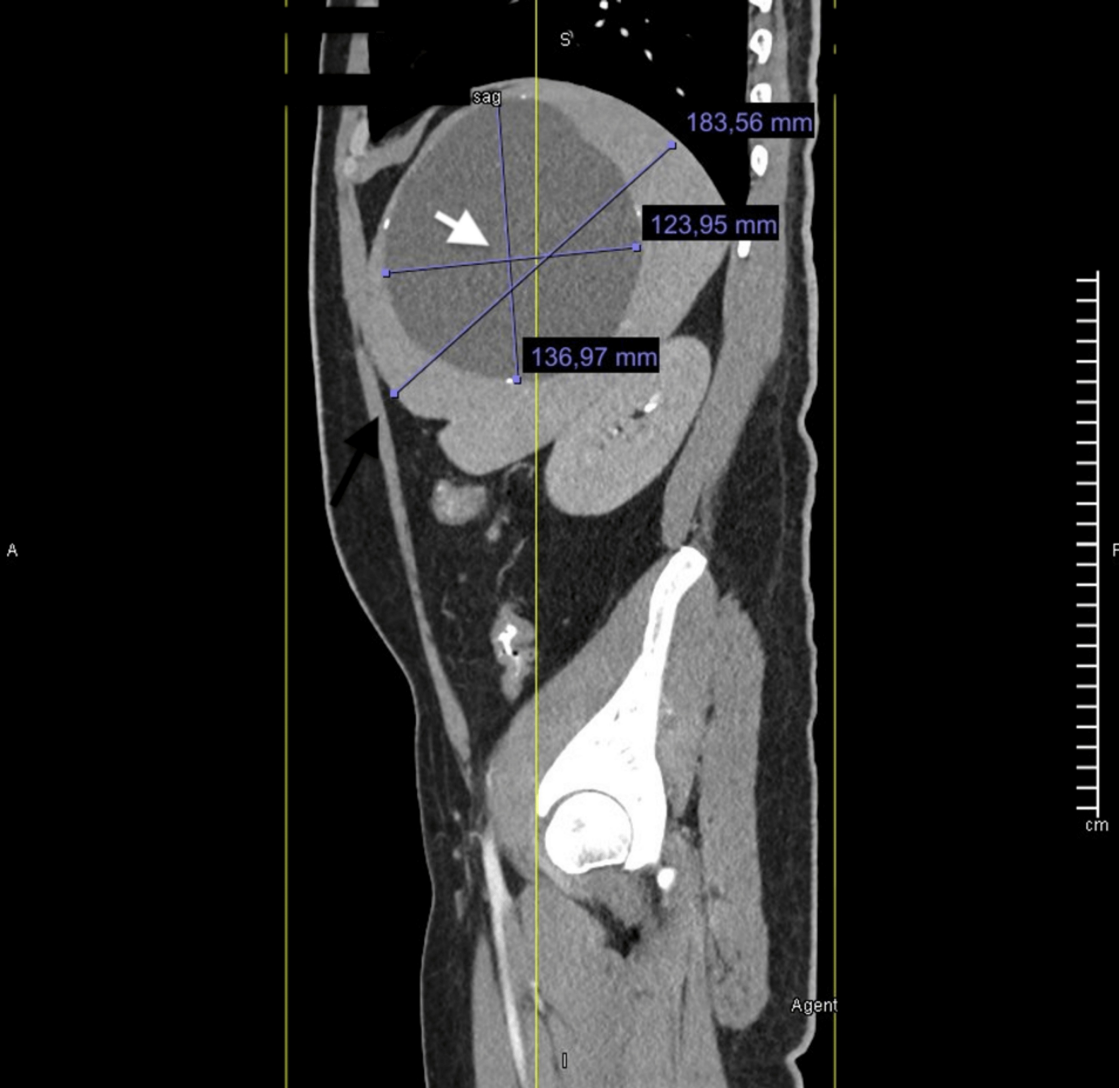
Contrast-enhanced computed tomography (sagittal view). Splenic measurement of 183.56 mm (black arrow). Splenic cyst measurement of 123.95 × 136.97 mm (white arrow).

Contrast-enhanced computed tomography (CT) showed splenomegaly secondary to a homogeneous, thin-walled cystic lesion with regular borders, no septa, and lamellar calcifications, measuring 129.33 × 138.78 × 136.97 mm, with a density of 16-19 HU. The spleen measured 170.74 × 183.63 × 187.56 mm, consistent with massive splenomegaly (Figures 1, 2, 3).

**Figure 4:**
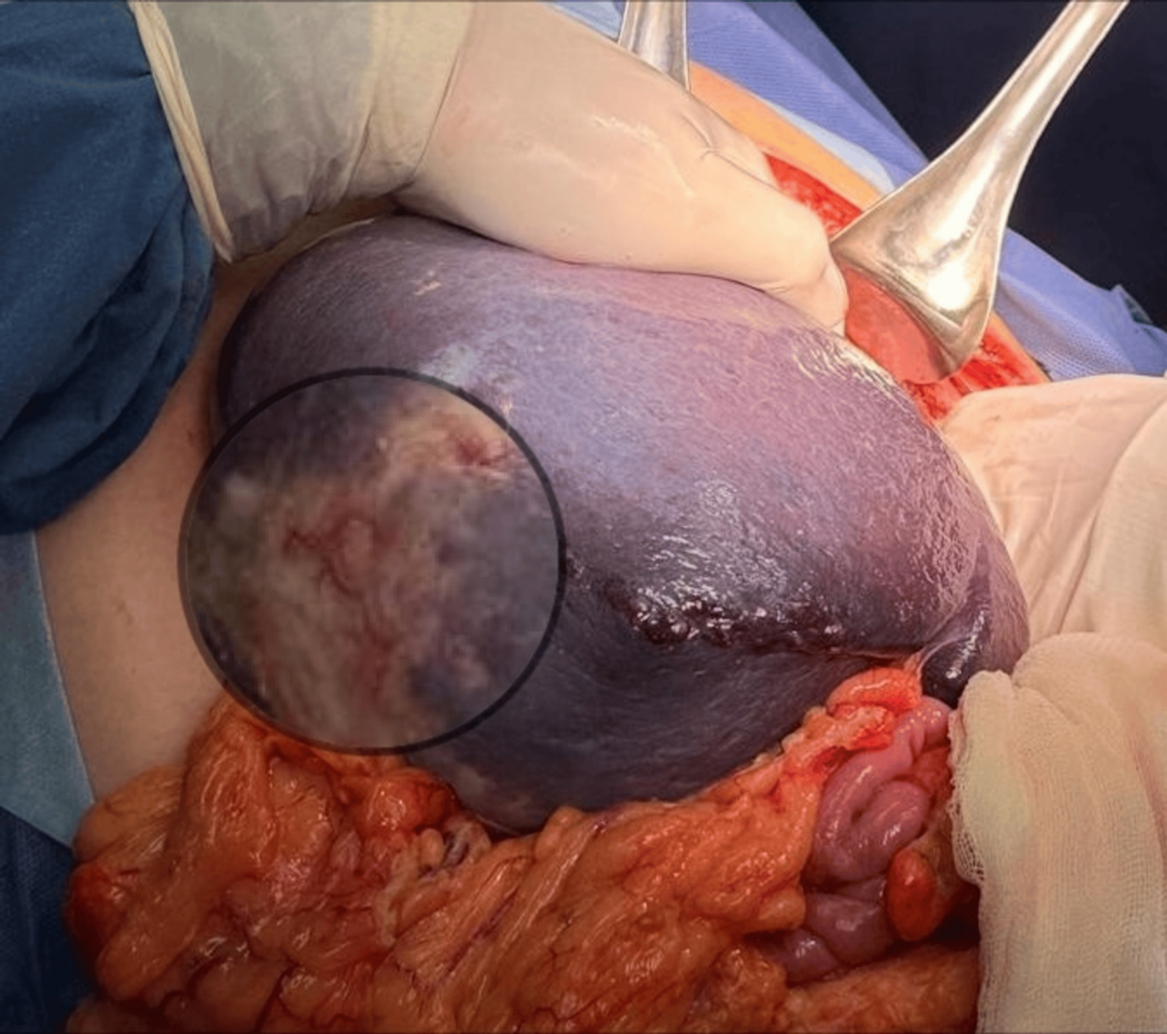
Exploratory laparotomy. The image shows the specimen obtained after open splenectomy. The circled area highlights the apparent cyst located in the upper pole, with a pale appearance.

The patient was scheduled for an elective open splenectomy. During surgery, a markedly enlarged spleen with a macronodular appearance and an apparent cyst at the upper pole was identified. The cyst had a pale surface and contained scant seropurulent fluid. The surgical specimen measured approximately 30 × 20 × 15 cm. Hemostasis was confirmed, and no lymphadenopathy or intra-abdominal implants were identified (Figure 4).

The postoperative period was uneventful, and the patient was discharged 48 hours after surgery. Histopathological examination revealed an intraparenchymal splenic cyst measuring 14 cm at its largest dimension, with extensive vascular congestion, marked vascular dilation within the parenchyma, and residual hyperplasia of the white pulp.

## Discussion

We present a rare case of giant splenomegaly secondary to an intraparenchymal splenic cyst without rupture or hemorrhage, successfully managed with conventional splenectomy without complications. As previously mentioned, there is a lack of high-level evidence establishing standardized diagnostic and therapeutic criteria.

Splenic cysts are usually diagnosed incidentally, particularly when small, and most commonly occur in the second decade of life. However, they may present with symptoms such as abdominal pain, early satiety, left shoulder pain, and compressive symptoms [3,4]. Our patient presented with similar symptoms, with fever being an atypical feature that prompted further investigation.

The diagnostic approach begins with imaging studies. Ultrasound is often the initial modality due to its availability and ability to provide a rapid presumptive diagnosis, although it is operator-dependent and limited in defining lesion topography. CT provides detailed information regarding lesion size, location, internal characteristics, and involvement of adjacent structures. Expert opinion suggests that CT or MRI should be obtained for surgical planning, and in large cysts, three-dimensional reconstruction may be useful to assess vascular relationships [3,4].

Definitive diagnosis requires histopathological and immunohistochemical analysis. In our case, pathology confirmed an intraparenchymal splenic cyst. Historically, total splenectomy was considered the standard treatment. However, due to the immunological role of the spleen and the risk of overwhelming post-splenectomy infection, spleen-preserving techniques have been increasingly adopted, particularly in pediatric and immunocompromised patients [5].

Non-parasitic cysts have been managed with percutaneous drainage and sclerotherapy; however, these approaches have fallen out of favor due to high recurrence and complication rates. Spleen-preserving surgical options include partial splenectomy and omental packing [5].

There remains controversy regarding surgical indications due to limited evidence. However, case series and reports suggest that cysts larger than 5 cm should be managed surgically due to the increased risk of rupture, hemorrhage, or infection [4,6-8].

In our case, due to the large size (>13 cm), intraparenchymal location, and risk of complications, an elective exploratory laparotomy with total splenectomy was performed, consistent with recommendations in the literature. Preoperative vaccination against encapsulated organisms (*Streptococcus pneumoniae, Neisseria meningitidis, and Haemophilus influenzae*) was administered.

## Conclusions

The management of splenic cysts should be individualized based on cyst type and patient characteristics. Cysts larger than 5 cm warrant special consideration due to the increased risk of complications such as rupture or hemorrhage. Whenever possible, spleen-preserving techniques should be considered to avoid post-splenectomy sepsis. However, total splenectomy remains a valid option in selected cases, particularly in large, intraparenchymal, or complex cysts, with appropriate vaccination against encapsulated organisms.

A major limitation of this study is that it represents a single case report. Due to the rarity of this condition and the lack of high-quality evidence, it remains difficult to establish standardized management criteria.
